# Evaluation of Febrile Seizures Focusing on the Diurnal Variation

**DOI:** 10.7759/cureus.71349

**Published:** 2024-10-13

**Authors:** Yoshifumi Miyagi, Yasunori Horiguchi, Yasuyuki Yano, Ryosuke Urabe, Atsushi Kitagawa, Hiroyuki Kato, Kentoku Kin

**Affiliations:** 1 Department of Pediatrics, Haibara General Hospital, Shizuoka, JPN; 2 Department of Pediatrics, International University of Health and Welfare, Atami Hospital, Atami, JPN

**Keywords:** children, circadian rhythm, convulsion, diurnal variation, febrile seizures

## Abstract

Background and objectives

Several studies have reported the diurnal variation of febrile seizures (FS) in children. However, it remains unclear whether there is a difference in diurnal variation depending on the types of FS. The present study aims to investigate whether simple FS or complex FS influences diurnal fluctuations.

Methods

In this single-facility retrospective study, Japanese pediatricians collected clinical data from 247 children with FS. We evaluated the diurnal occurrence of FS using medical files. The Kolmogorov-Smirnov test was used to assess differences in distribution by classifying days into four six-hour time periods: night (0:00-6:00), morning (6:00-12:00), afternoon (12:00-18:00), and evening (18:00-24:00).

Results

In the simple FS group, the highest rate was observed in the evening (35.16%), followed by the afternoon (29.69%), with the lowest rate in the morning (14.06%). In the complex FS group, the highest rates were observed in both the afternoon (30.95%) and evening (30.95%), with the lowest rate at night (16.67%). The distributions of simple FS and complex FS were significantly different.

Conclusion

FS exhibited different diurnal variations depending on the type of FS. Clinicians' recognition of these findings will aid in treatment.

## Introduction

Febrile seizures (FS) represent the most common neurologic disorder affecting infants and young children. They are age-dependent occurrences, affecting approximately 2% to 14% of children worldwide [1,2]. FS mainly occurs in children aged 6 to 60 months. They typically manifest as diseases with paroxysmal episodes, both convulsive and nonconvulsive, associated with a fever of 38°C or higher. Notably, they occur in the absence of other underlying seizure-provoking causes or diseases, such as central nervous system infections, electrolyte abnormalities, drug withdrawal, trauma, genetic predisposition, or known epilepsy [3].

Complex FS is defined by the presence of one or more of the following three criteria, while those not meeting these criteria are classified as simple FS [4]: 1) elements indicative of focal seizures (partial seizures); 2) seizures lasting longer than 15 minutes; and 3) multiple recurrent seizures within a single fever episode, typically occurring within a 24-hour period.

The exact pathophysiology of FS is unknown, but it is thought to be caused by a combination of genetic predisposition and environmental factors (fever and its cause) [5]. We thought it necessary to investigate the differences in seizure type as one of these factors. Several reports have highlighted diurnal variations in FS occurrences [6-10]. However, these studies have primarily focused on specific subgroups, such as simple FS [6-8], complex FS [9], or first attacks [10], limiting their generalizability. Additionally, none of these studies have explored the relationship between FS types and diurnal variation. The present study aims to investigate whether simple FS or complex FS influences diurnal fluctuations.

## Materials and methods

Study design and subjects

The study protocol adhered to the principles outlined in the Declaration of Helsinki and received approval from the Ethics Committee of the International University of Health and Welfare, Atami Hospital (approval date: May 27, 2024; approval number: 24-A-247). This retrospective cohort study analyzed 247 patients with FS treated between 2015 and 2023 at the Department of Pediatrics, International University of Health and Welfare, Atami Hospital, Japan. We retrospectively examined parameters such as age, sex, temperature, seizure onset time, and the presence of partial factors, prolonged duration, and recurrence based on patient records. The onset time was obtained by re-examining the medical records, based on reports by caregivers and eyewitnesses.

Inclusion and exclusion criteria

Infants and children who presented to the emergency department with cramps accompanied by a fever of 38°C or higher were included in the study. Children with a history of afebrile seizures or epilepsy were excluded, as were those with other diseases or conditions that would satisfy acute symptomatic seizures, such as intracranial infections.

Definitions and clinical data

Simple FS was defined as a generalized seizure lasting less than 15 minutes without recurrence within one episode, while complex FS met criteria including focal seizures, durations exceeding 15 minutes, or recurrent seizures within one episode. We assessed the proportion of each FS type and the occurrence of complex FS features. Additionally, body temperature at the onset of FS was illustrated using a histogram. Body temperature measurements closest to the time of onset, including those at hospital arrival, ambulance arrival, or just before onset at home, were considered. Seizures with temperatures between 37°C and 38°C were included if they exceeded 38°C immediately after onset. Regarding FS onset, we divided each day into 24 segments to show the hourly onset count, then classified days into four six-hour time periods (night, morning, afternoon, and evening) to address the issue of insufficient samples per hour in the 24-hour distribution.

Outcome measures

The distributions were statistically analyzed and compared to equal distributions. In addition, to identify differences in the diurnal variation between simple and complex FS, we statistically compared the distributions of the two groups by dividing them into four groups.

Statistics

Age, sex, body temperature, seizure duration, and number of seizures during one episode were statistically evaluated. One-way ANOVA was used for normally distributed continuous variables, the Kruskal-Wallis test was used for non-normally distributed continuous variables, and the chi-square test was used for categorical variables. The Kolmogorov-Smirnov test was used to assess differences in distribution. Data manipulation and modeling were conducted using Python 3 (version 3.6.9). All tests were two-tailed, with P < 0.05 considered significant.

## Results

Population demographics

**Figure 1:**
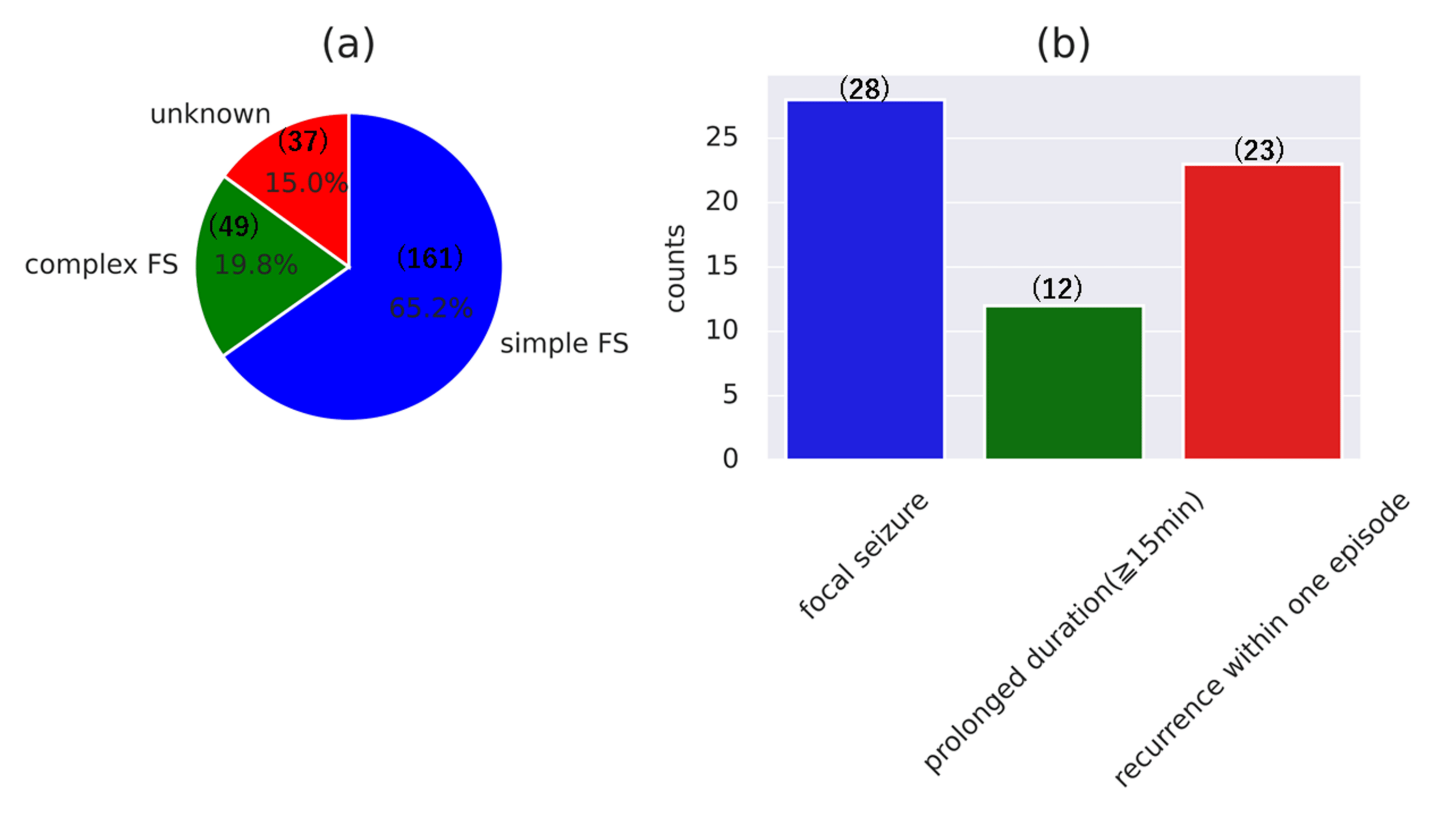
Demographics of febrile seizures (a) The ratio of simple FS and complex FS. (b) Number and proportion of features in complex FS. Numbers in brackets indicate the number of cases. FS: febrile seizures

A total of 247 patients between three months and nine years of age were included in the study, comprising 152 (61.5%) boys and 95 (38.5%) girls, with a mean age of 2.67 ± 1.76 years. The mean temperature of the patients was 39.22 ± 0.88°C. The mean duration of seizures was 3.88 ± 4.86 minutes. Simple FS are more prevalent than complex FS and are characterized by generalized seizures lasting less than 15 minutes without recurrence within the same fever episode. Due to insufficient information for classification, 15.0% of cases were labeled as unknown. The distribution was 65.2% for simple FS and 19.8% for complex FS (Figure 1a). In our study, focal seizures accounted for 11.34%, prolonged seizures (≥15 minutes) for 4.86%, and recurrence for 9.31% (Figure 1b).

**Figure 2:**
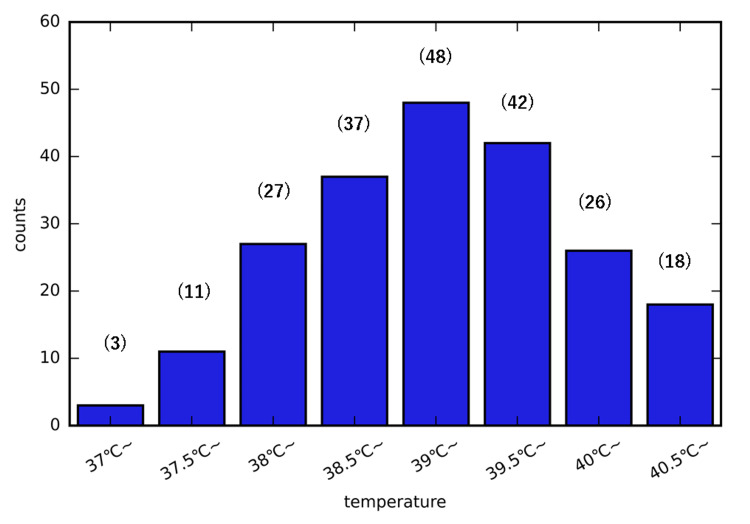
Histogram of temperature at onset of febrile seizures Numbers in brackets indicate the number of cases.

At the onset, 6.61% of body temperatures were below 38°C, but the majority exceeded 38°C (Figure 2).

Diurnal variation of FS

**Figure 3:**
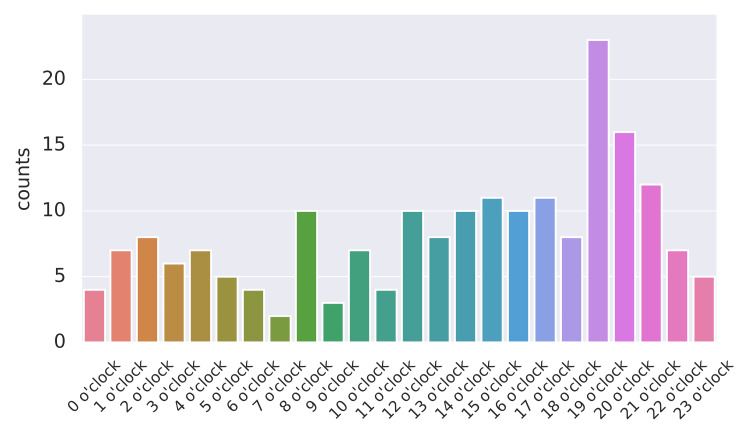
Hourly data on the occurrence of febrile seizures The diurnal variation was highest between 19:00 and 20:00. Kolmogorov-Smirnov test was used to assess differences in distribution, and P < 0.05 was considered significant. Statistical test for even distribution (P < 0.05).

Regarding the time of onset, a higher frequency of onset was observed post-meridian compared to ante-meridian, peaking between 19:00 and 20:00 in the 24-hour divided table. This fluctuation was statistically significant compared to an even distribution (P < 0.05) (Figure 3).

**Figure 4:**
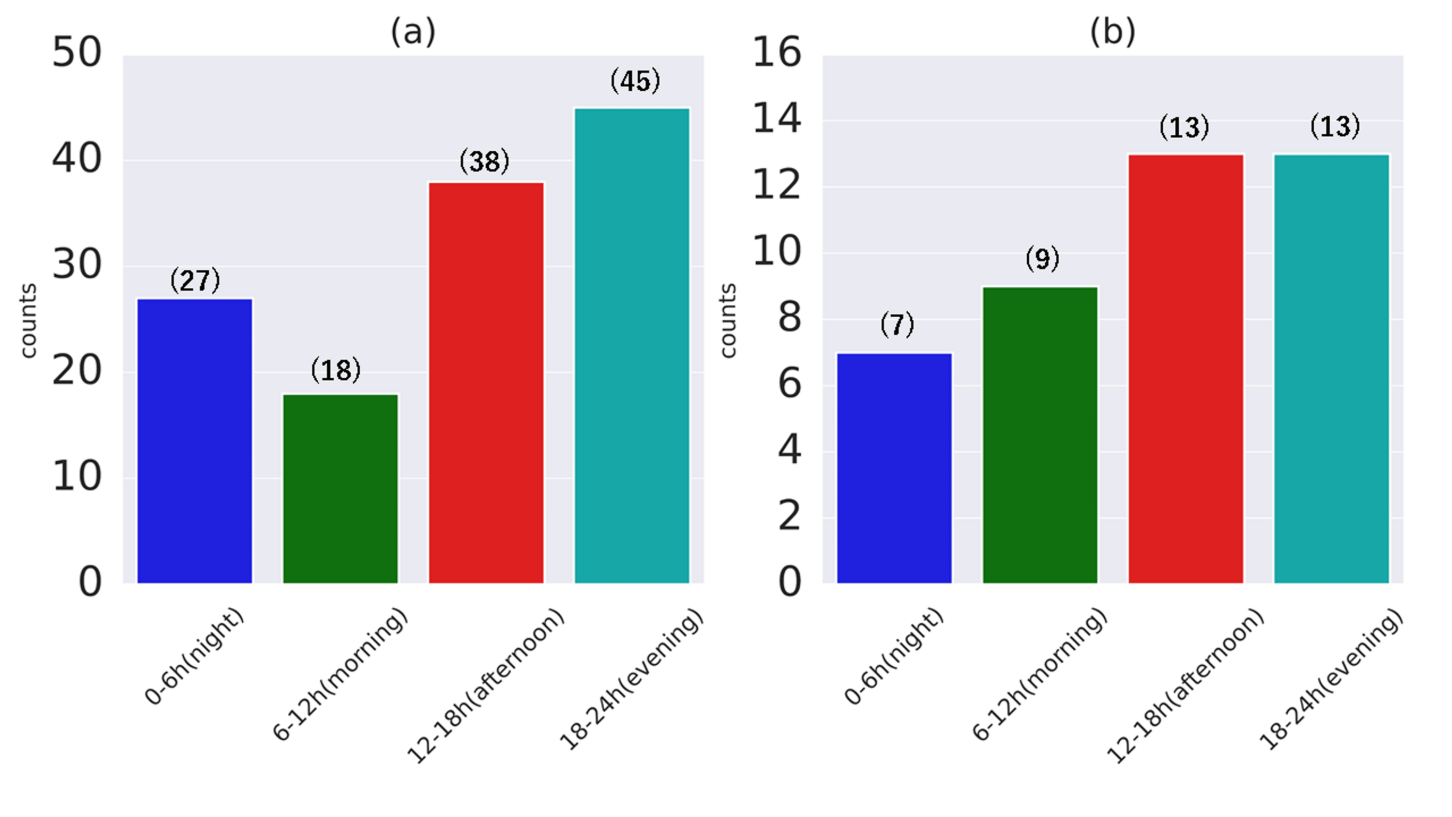
Histogram of the number of febrile seizures dividing the day into four parts The occurrence of FS subdivided into four six-hour periods in (a) simple FS and (b) complex FS. Numbers in brackets indicate the number of cases. Kolmogorov-Smirnov test was used to assess differences in distribution, and P < 0.05 was considered significant. The difference in distribution between simple FS and complex FS was confirmed (P < 0.05). FS: febrile seizures

When the day was divided into four groups: night (0:00-6:00), morning (6:00-12:00), afternoon (12:00-18:00), and evening (18:00-24:00). In the simple FS group, the highest rate was observed in the evening group (35.16%), followed by the afternoon (29.69%), with the lowest rate (14.06%) in the morning (Figure 4a). In complex FS, the highest rate was observed in both the afternoon and evening (30.95%), with the lowest rate (16.67%) at night (Figure 4b). Additionally, a significant difference in distribution between simple FS and complex FS was confirmed (P < 0.05).

In these four groups, we evaluated differences in age, sex, temperature, seizure duration, and the number of seizures within one episode. However, no significant differences were observed among these factors (Table 1).

**Table 1:** Comparison of features of FS between the four categories of time of day P < 0.05 was considered significant. ^†^: Chi-squared test; ^§^: one-way ANOVA; ^¶^: Kruskal-Wallis test

	0-6 hours (night)	6-12 hours (morning)	12-18 hours (afternoon)	18-24 hours (evening)	P-value
	N = 37	N = 30	N = 60	N = 71	
Categorical variable					
Sex (male (%))	26 (70)	17 (57)	40 (67)	39 (55)	0.32^†^
Continuous variable (mean±std)					
Temperature (℃)	39.2 ± 0.97	39.1 ± 0.94	39.2 ± 0.88	39.4 ± 0.84	0.49^§^
Continuous variable (median (IQR))					
Age (year)	1.7 (1.2, 2.6)	2.9 (1.2, 4.9)	2.4 (1.7, 3.6)	2.3 (1.4, 3.6)	0.25^¶^
seizure duration (min)	4.0 (1.8, 5.0)	2.0 (1.0, 5.0)	3.0 (1.0, 5.0)	2.0 (1.0, 5.0)	0.36^¶^
number of seizure within one episode (times)	1.0 (1.0, 1.0)	1.0 (1.0, 1.0)	1.0 (1.0, 1.0)	1.0 (1.0, 1.0)	0.15^¶^

## Discussion

This paper provides evidence supporting the existence of a diurnal cycle in FS. However, the cause of this diurnal variation remains elusive. Potential explanations include the influence of melatonin or body temperature fluctuations.

In a study by Smolensky et al. (2023) reporting on febrile seizures overall, a time series meta-analysis examined the 24-hour pattern of FS onset and found a four-fold difference between the peak at 18:04 and the trough at 06:00 of the seizure [11]. For complex FS in particular, Yamaguchi et al. (2018) found that the frequency of CFS increased during the day and in the evening, with 18:00 being about five times greater than 02:00 [10]. The peak times may differ slightly, but these reports are consistent with our analysis results.

The diurnal pattern of FS is thought to derive from multiple circadian rhythms, particularly the cytokines that comprise the thermogenic inflammatory pathway and the action of melatonin, which affects the excitation level of central neurons and helps regulate body temperature [11]. Several articles suggest that melatonin may possess anticonvulsant effects [12,13]. Additionally, lower melatonin levels have been reported in FS patients compared to healthy children [14]. Conversely, a separate study found no significant differences in salivary melatonin levels between FS patients and control individuals, leading to the conclusion that melatonin may not play a significant role in simple or complex FS [15]. This discrepancy suggests that the role of melatonin in FS is controversial.

Regarding temperature, it's well documented that body temperature exhibits diurnal variation [16]. Given that evening and afternoon hours already experience a physiological increase in body temperature, it's not surprising that FS occurrences peak during these times. Furthermore, studies have suggested that the absolute temperature itself may be more critical than the rate of temperature rise, as both high-frequency and low-frequency groups exhibited similar temperatures at the onset of FS [7]. In our study, we did not observe a significant difference in body temperature, which aligns with existing literature. However, accurately measuring body temperature at the onset of FS poses challenges. Additionally, our report noted variations in the threshold value, ranging from 37°C to over 41°C. Therefore, the influence of body temperature remains a subject of debate.

FS, the most common form of seizure in childhood, arises from multifactorial causes. Moreover, the occurrence of seizures in patients with epilepsy may be influenced by circadian rhythms. Additionally, different diurnal variations have been observed depending on the type of FS. We hypothesized that the diurnal variation could be influenced by the seizure type. Studies in epilepsy have reported various diurnal patterns based on seizure type [17,18]. For instance, generalized seizures typically peak between 6 am and 12 pm, temporal lobe seizures between 9 pm and 9 am, frontal lobe seizures between 12 am and 6 am, parietal lobe seizures between 6 am and 9 am, and occipital lobe seizures between 9 am and noon and 3 pm and 6 pm [17]. Furthermore, clonic, tonic, and tonic-clonic seizures exhibit peak activity between 6:00 and 9:00 using a three-hour bin interval [18]. Although FS generally manifests as generalized clonic, tonic, or tonic-clonic seizures, the rhythmic patterns observed in other types of epilepsy differ from FS. It's essential to consider that FS itself may have its own cycle. Additionally, if there are changes in fluctuations due to geographical location, simple FS and complex FS may demonstrate different diurnal patterns, particularly as complex FS involves focal seizures. We attempted to investigate generalized seizures and focal seizures in FS to explore the impact of seizure locality on fluctuations. However, due to the small sample size in our study, we were unable to do so. Further research is warranted in this area.

The main strength of our study lies in the statistical analysis of the differences in diurnal variation depending on the types of FS. We demonstrated distinct diurnal patterns between complex and simple FS. However, our study also possesses certain limitations. The relatively small sample size, inherent to a retrospective study conducted at a single center, is one limitation. Additionally, the reliance on interviews with parents and witnesses for determining the time of seizure onset introduces potential inaccuracies. Furthermore, the lack of accurate information further reduced the number of subjects. Larger patient cohorts are necessary for future studies to address these limitations.

For clinicians, awareness of these findings can inform treatment decisions. While the risk of epilepsy in the future is slightly elevated compared to the general population, the prognosis for most children with FS is excellent. However, parents of children with FS should be educated about the nature of convulsions. Parental anxiety can be significant, and thus, appropriate education and emotional support are crucial. Knowledge of significant differences in risk by time of day of FS may help to gauge the appropriate timing of preventive interventions. Understanding individual diurnal seizure patterns can also serve as a guide for treatment approaches.

## Conclusions

FS exhibited different diurnal variations depending on whether they were classified as simple or complex. Proper characterization of diurnal seizure patterns is important as it serves as a guide for treatment approaches. These findings should be recognized by clinicians when considering treatment strategies.
